# High salt induces anti-inflammatory MΦ2-like phenotype in peripheral macrophages

**DOI:** 10.1016/j.bbrep.2016.05.009

**Published:** 2016-05-12

**Authors:** Suneetha Amara, Margaret Whalen, Venkataswarup Tiriveedhi

**Affiliations:** a Department of Medicine, Mercy Hospital, St Louis, MO, United States; b Department of Chemistry, Tennessee State University, TN, United States; c Department of Biological Sciences, Tennessee State University, TN, United States

**Keywords:** Macrophage, Inflammation, Cancer, Cytokine, INOS, Arg-1

## Abstract

Macrophages play a critical role in inflammation and antigen-presentation. Abnormal macrophage function has been attributed in autoimmune diseases and cancer progression. Recent evidence suggests that high salt tissue micro-environment causes changes in macrophage activation. In our current report, we studied the role of extracellular sodium chloride on phenotype changes in peripheral circulating monocyte/macrophages collected from healthy donors. High salt (0.2 M NaCl vs basal 0.1 M NaCl) treatment resulted in a decrease in MΦ1 macrophage phenotype (CD11b^+^CD14^high^CD16^low^) from 77.4±6.2% (0.1 M) to 29.3±5.7% (0.2 M, p<0.05), while there was an increase in MΦ2 macrophage phenotype (CD11b^+^ CD14^low^CD16^high^) from 17.2±5.9% (0.1 M) to 67.4±9.4% (0.2 M, p<0.05). ELISA-based cytokine analysis demonstrated that high salt treatment induced decreased expression of in the MΦ1 phenotype specific pro-inflammatory cytokine, TNFα (3.3 fold), IL-12 (2.3 fold), CCL-10 (2 fold) and CCL-5 (3.8 fold), but conversely induced an enhanced expression MΦ2-like phenotype specific anti-inflammatory cytokine, IL-10, TGFβ, CCL-17 (3.7 fold) and CCR-2 (4.3 fold). Further high salt treatment significantly decreased phagocytic efficiency of macrophages and inducible nitric oxide *syn*thetase expression. Taken together, these data suggest that high salt extracellular environment induces an anti-inflammatory MΦ2-like macrophage phenotype with poor phagocytic and potentially reduced antigen presentation capacity commonly found in tumor microenvironment.

## Highlights

• High salt induced macrophage switch from MΦ1 to MΦ2-like phenotype.
• High salt induced anti-inflammatory MΦ2-specific cytokine profile.
• Reduced phagocytic efficiency upon high salt treatment.
• Inhibition of iNOS activity following high salt stimulation.

## 1 Introduction

Macrophages have an important role in innate immunity mediated inflammation and host defense [1]. Transformation of different phenotypes of macrophages regulate the initiation, development, and cessation of inflammatory diseases [2]. While, classically activated macrophages (MΦ1) comprise immune effector cells with an acute inflammatory and phagocytic phenotype [3]. Conversely, alternatively activated MΦ2-macrophages are anti-inflammatory and aid in inhibition of immune-damage and immune-tolerance [4]. A variety of external signals, such as, microbes, damaged tissue and infiltrating lymphocytes have been shown to play an important role in the preferential MΦ1 versus MΦ2 macrophage activation. In the tumor micro-environment, among the innate and adaptive immune cells recruited to the tumor site, macrophages are particularly abundant and are present at all stages of tumor progression [5]. Clinical studies and experimental mouse models indicate that these macrophages generally play an anti-inflammatory and pro-tumoral role [6]. Specifically, the tumor associated macrophages (TAM) have an MΦ2 phenotype with reduced antigen presentation capacity, thereby possibly leading to evasion of cancer immuno-elimination [7]. Further recent evidence suggests that high levels of TAM are often correlated with tumor metastasis and poor prognosis [5].

The high sodium content of mammary adenocarcinomas has been shown to be significantly higher than the normal lactating mammary epithelium [8]. Interestingly, recent studies by Jantsch et al. [9], suggest that skin infiltrating macrophages undergo phenotype changes [9]. However, the dermal micro-environment is known to have high sodium tonicity. These dermal resident macrophages have been indicated to sense the interstitial electrolyte composition and subsequently upregulate various transcription factor possibly resulting in their activation and phenotype changes [10]. Similarly high salt is considered to influence the macrophage activation in lung [11] and bone-marrow derived mouse macrophages[11]. However, currently there is limited evidence to demonstrate the direct role of high salt on macrophage activation. Peripheral blood mononuclear cells (PBMC) derive from myelomonocytic precursors in the bone marrow. Myelomonocytes circulate in the blood for approximately 72 h and then migrate to tissues to become macrophages [12]. The pattern of in vitro modulation of cell surface antigens in PBMC in many respects resembles the pattern seen in tissue macrophages [12]. As salt changes have been noted in cancer development [13], and these sites have higher frequency of alternatively activated MΦ2-macrophages, we hypothesized that high salt directly induces MΦ2-macrophage activation. In our current study, using in vitro macrophage cultures we directly determined the role of high sodium chloride concentration on macrophage phenotype changes.

## 2 Materials and methods

### 2.1 Monocyte/Macrophage isolation and culture

Peripheral blood mononuclear cell isolation and cell culture: Peripheral blood mononuclear cells (PBMC) leukocyte filters (PALL- RCPL or RC2D) obtained from the Red Cross Blood Bank Facility (Nashville, TN, USA) and isolated as previously described [14]. PBMC were retrieved from the filters by back-flushing of the leukocyte filter and separating the PBMC (peripheral blood mononuclear cells) on ficoll-Hypaque gradient as previously described [15]. These PBMCs were cultured in for 72 h in a glass culture flask and adherent cells, which were considered in vitro equivalent of macrophages, were subjected to various treatment conditions for 48 h. For high salt studies, the cells were treated with 0.1–0.3 M NaCl (final concentration). To account for the osmotic tonicity with high solute concentration equimolar mannitol (0.1–0.2 M mannitol+0.1 M NaCl) was used as negative control. All chemicals unless mentioned were obtained either from Sigma-Aldrich (St Louis, MO) or Fisher (Pittsburgh, PA).

### 2.2 MTT cell viability assay

Cell viability was measured by tryphan blue dye exclusion (Sigma Aldrich, MO) and MTT assay (Life technologies, Grand Island, NY) as previously described [16]. The assay was performed as per manufacturer provided instructions and plates were read at 562 nm by the plate reader (EMax Plus spectrophotometer, Molecular Devices, Sunnyvale, CA). Viability was calculated as percentage compared to untreated cells in basal conditions.

### 2.3 Flow cytometry

The CD11b positive cells were isolated from PBMC by positive isolation using immunomagnetic beads (Miltenyi, San Diego, CA). Antibodies used for flow cytometry consisted of: CD11b-FITC, CD14-PE, and CD16^−^PerCP (Santa Cruz), CD25-PE, Foxp3-PE (eBiosciences, San Diego, CA). Samples were analyzed using a FACS Calibur/LSRII flow cytometer (Becton Dickinson, Franklin Lakes, NJ) and cell sorting was performed with a Vantage cell sorter (Becton Dickinson, Franklin Lakes, NJ). Data were analyzed using BD FACSDiva software. Gates were set according to isotype controls.

### 2.4 Enzyme-linked immunosorbant assay

The secretory extracellular cytokines in the cell supernatant was quantitatied by ELISA. Chemokine/Cytokine ELISA was performed as per the manufacturer's protocol (eBiosciences, San Diego, CA). Given the limitation of the detection, the supernatant was diluted 1:1000 and quantified with a standard curve using the manufacturer provided standards. Detection at 450 nm was performed using EMax Plus spectrophotometer and data analysis was carried out using software provided by the manufacturer (Molecular Devices, Sunyvale, CA).

### 2.5 Phagocytosis assay with RBC-lysis and E.coli uptake

For phagocytosis and cell lysis, we have utilized CytoSelect™ 96-Well Phagocytosis RBS substrate Assay (CBA-220, Cell Biolabs Inc, San Diego, CA). Briefly, the lytic ability of macrophages towards sheep erythrocytes was analyzed in a 96 well plate as per manufacturer's protocol with RBC to macrophage ratio maintained at 10:1. The specific lysis was analyzed by calorimetric analysis at 630 nm.

For phagocytosis and engulfment assay, we have utilized Vybrant™ Phagocytosis Assay Kit (V-6694, ThermoFisher Scientific, Waltham, MA). Briefly, internalization of fluorescent (FITC) labeled killed E-coli (K12-strain) by macrophages was analyzed in a 96 well plate as per manufacturer's protocol with killed bacteria to macrophage ratio maintained at 20:1. Internalization was these particles was analyzed on fluorescence plate reader at excitation/ emission of 480/520 nm, respectively.

### 2.6 Western blot analysis and quantitative RT-PCR

Specific protein determination studies were performed by Western-blot on the total proteins were extracted from cells as described earlier [17]. Protein concentration was determined with a Bradford's assay kit from Bio-Rad (Hercules, CA). All primary and secondary Abs were obtained from Santa Cruz Biotech (Dallas, TX). The membranes were developed using the chemiluminescence kit (Fischer Sci, Pittsburgh, PA) and analyzed on using Bio-Rad Universal Hood II (Hercules, CA). Morphometric analysis was done using the software provided by the company. Expression profiles of intracellular signal genes in the CD4+T-cells isolated from PBMCs were analyzed using the FAM-labeled quantitative RT-PCR primers for IL-10, IFN-γ, T-bet and FoxP3 (Applied Biosystems, Foster City, CA) as per the manufacturer's recommendation and described earlier [18].

### 2.7 NO/ROS/RNS analysis

The analysis of the nitric oxide (NO), reactive oxygen and reactive nitrogen species (AbCam, Cambridge, MA) was performed in the cell lysate under various assay conditions as per manufacturer's instructions. The data analysis was performed based on a standard curve obtained using the positive controls provided by the manufacturer.

### 2.8 Statistical analysis

Data are expressed as mean ± SEM from four independent studies. Statistical differences between means were analyzed using a paired or unpaired Student's *t* test. A *p-value* of less than 0.05 was considered significant. All data analysis was obtained using Origin 7 software (Origin Labs, Northampton, MA) or GraphPad5 (Graph Pad Software, LaJolla, CA).

## 3 Results

### 3.1 High salt induced macrophage switch from MΦ1 to MΦ2-like phenotype

**Fig. 1:**
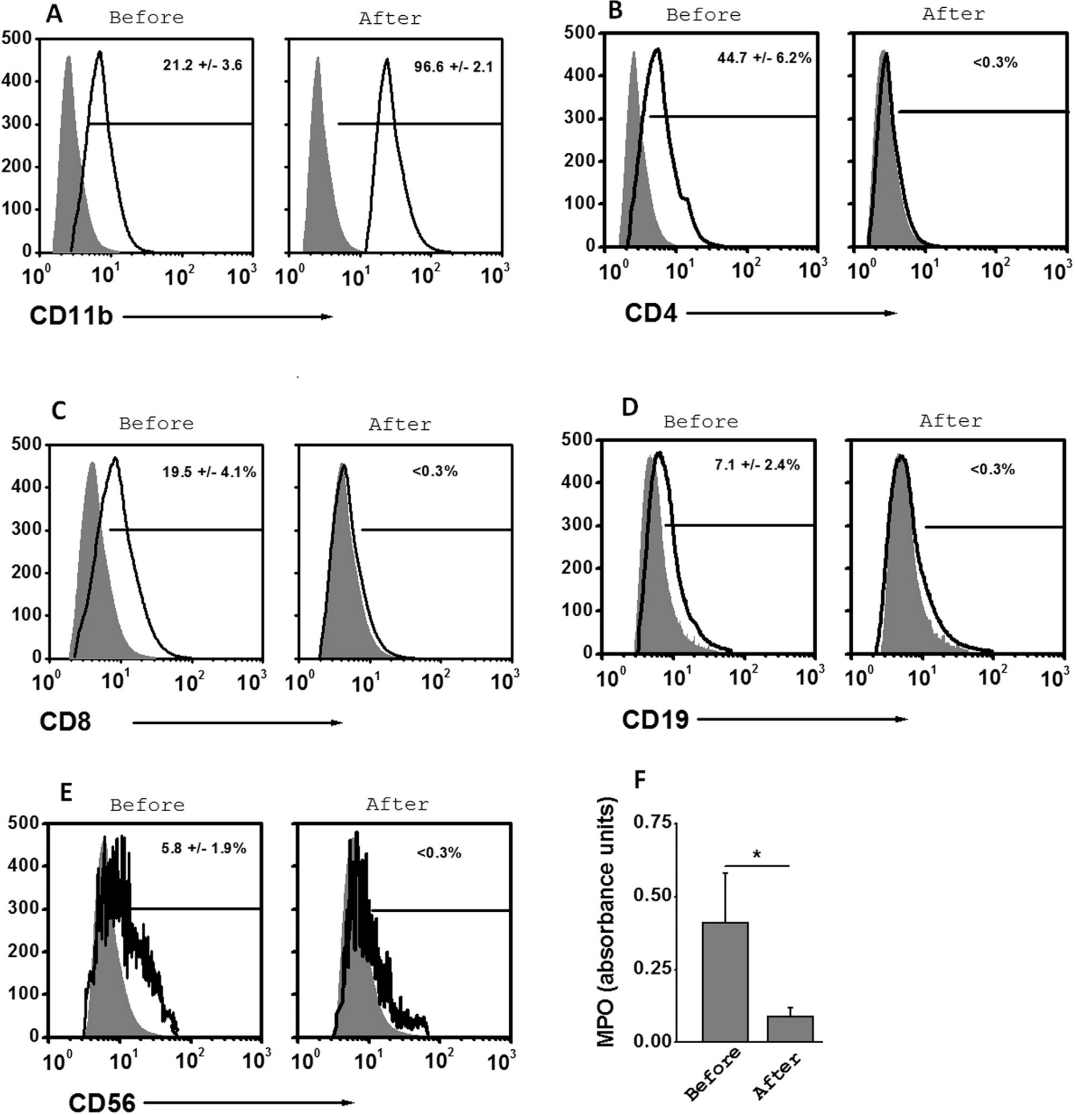
Isolation of glass-adherent CD11b+ macrophages from peripheral blood mononuclear cells (PBMCs). The PBMCs were cultured in a glass dish and cell specific phenotypes were analyzed. “Before’ refers to the phenotype analysis in the freshly collected PBMCs; ‘After’ refer to the phenotype analysis of the glass-adherent cells following 72 h culture and removal of the supernatant and non-adherent cells. After 72 h, the adherent cells were washed three times with fresh RPMI media and used in our further studies to determine the salt-effects. Increased macrophage phenotype (CD11b, A) in the adherent cells, along with decreased CD4+T cells (B), CD8+T cells (C), CD19+B cells (D), CD56+NK cells (E) and MPO (F, myeloperoxidase, leukocyte marker) in the adherent cells. Data represented mean values ± SEM from five independent experiments. Student-*t*-test performed for statistical analysis (significance p<0.05).

**Fig. 2:**
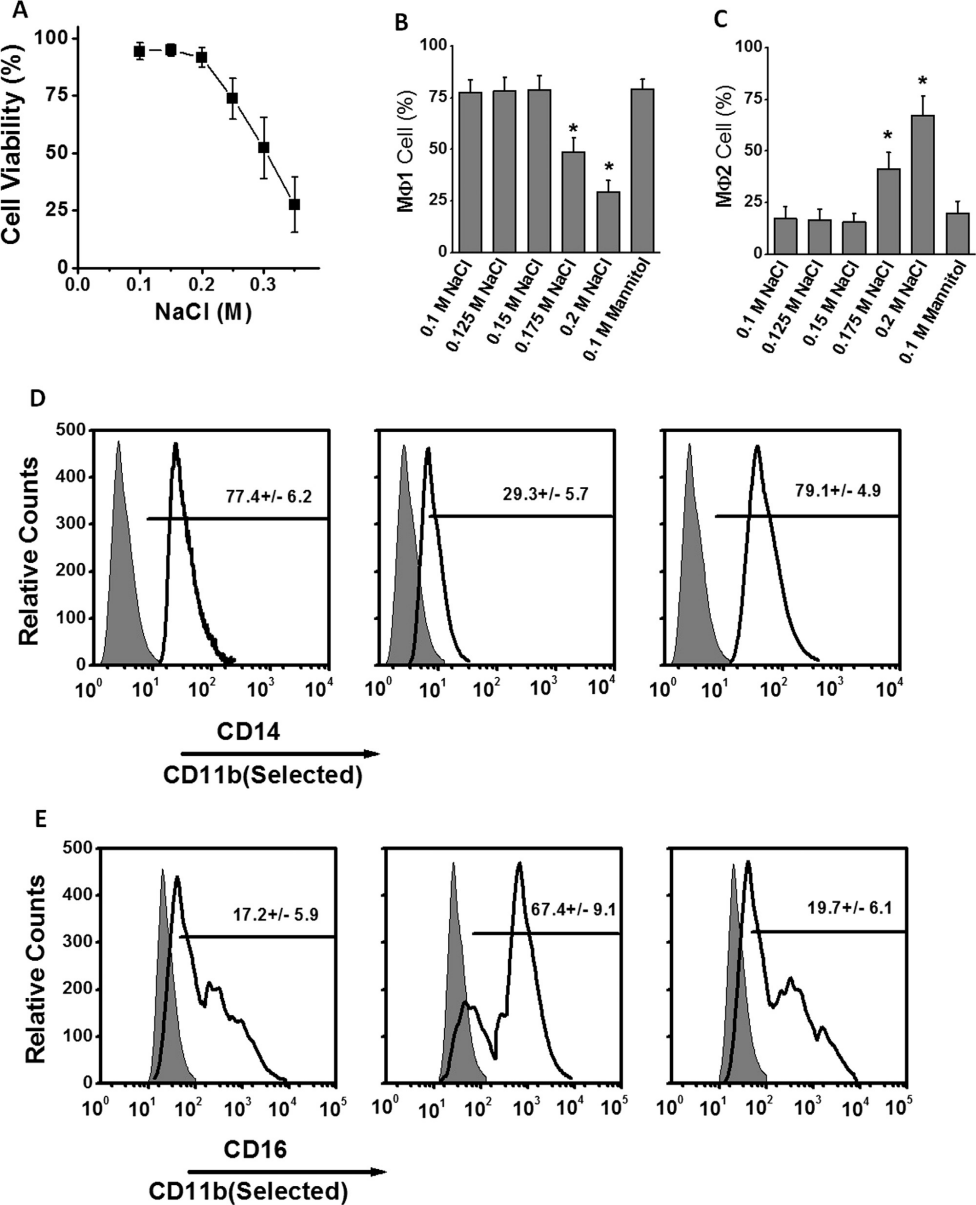
High salt induced macrophage phenotype switch from MΦ1 to MΦ2 in the adherent-PBMCs following treatment with varying salt concentration (0.1–0.3 M NaCl) and equimolar mannitol (0.1 M mannitol+0.1 M NaCl). The basal concentration of NaCl in cuture media is 0.1 M. NaCl concentration below 0.1 M is not viable for cell culture. (A) Cell viability analysis of adherent cells from PBMC cultured for 72 h and latter subjected to various treatment condition mentioned above for 48 h. Frequency of CD11b^+^CD14^+^CD16^low^ cells (B), and CD11b^+^CD14^low^CD16^+^ cells (C) following various treatment conditions. Flow cytometry analysis for CD14 (D) and CD16 (E) in the CD11b positively selected cells following various treatment conditions mentioned above. Data represented mean values ± SEM from five independent experiments. Student-*t*-test performed for statistical analysis (significance p<0.05).

High sodium chloride (NaCl) concentration in the tissue micro-environment has been suggested to induce macrophage changes. To specifically determine the effect of high sodium chloride on peripheral circulating macrophages collected from healthy human subjects, we have performed dose dependent studies under varying (0.1–0.3 M) sodium chloride concentrations. Initially, we have isolated macrophages from PBMCs by culturing the PBMCs in a glass culture dish and removing all non-adherent cells. While lymphocytes and other cells in the PBMCs are non-adherent only macrophages are the adherent phenotype. As shown in Fig. 1, following isolation of the adherent PBMCs, the frequency of CD11b+cells, a generic marker of macrophage-phenotype, increased from 21.2–96.6%, thus suggesting that all of the isolated adherent PBMCs were indeed predominantly macrophages. Conversely, all other cells in the PBMCs such as CD4+T cells, CD8+T cells, CD19+B-cells, CD56+ NK cells and leukocytes (as studied by myeloperoxidase, MPO assay) accounted for less than 0.3%. We have utilized these glass-adherent macrophages in our further studies to determine the potential effects of salt on the macrophage inflammatory phenotype change. As shown in Fig. 2(A), concentrations above 0.25 M resulted in significant (up to >50%) decrease in cell viability. Therefore, in all our subsequent studies we have used 0.2 M NaCl to represent high salt condition, while 0.1 M NaCl was considered regular salt extracellular milieu. Following treatment of freshly collected human macrophages with high salt for 24 h, there was a decrease in MΦ1 (Fig. 2(B), (D)) macrophage phenotype (CD11b^+^CD14^+^CD16^low^) from 77.4±6.2% (0.1 M) to 29.3±5.7% (0.2 M, p<0.05). Conversely, under similar treatment conditions there was an increase in MΦ2 (Fig. 2(C), (E)) macrophage phenotype (CD11b^+^CD14^low^CD16^+^) from 17.2±5.9% (0.1 M) to 67.4±9.4% (0.2 M, p<0.05). To rule out a possible role of intracellular volume and solute tonicity as a potential cause of the macrophage phenotype switch, we have performed experiments following treatment with equi-molar mannitol (0.1 M NaCl +0.1 M mannitol). As shown in Fig. 2, equi-molar mannitol did not induce any significant change in the macrophage phenotype over 0.1 M NaCl treatment conditions. Further, it is important to note a trending pattern between 0.15 M and 0.2 M NaCl concentration, suggesting that salt concentrations below 0.15 M did not induce any phenotype changes in the macrophages. Therefore, these data strongly suggest that the observed phenotype switch is due to high salt treatment.

### 3.2 High salt induced MΦ2 specific cytokine profile

**Fig. 3:**
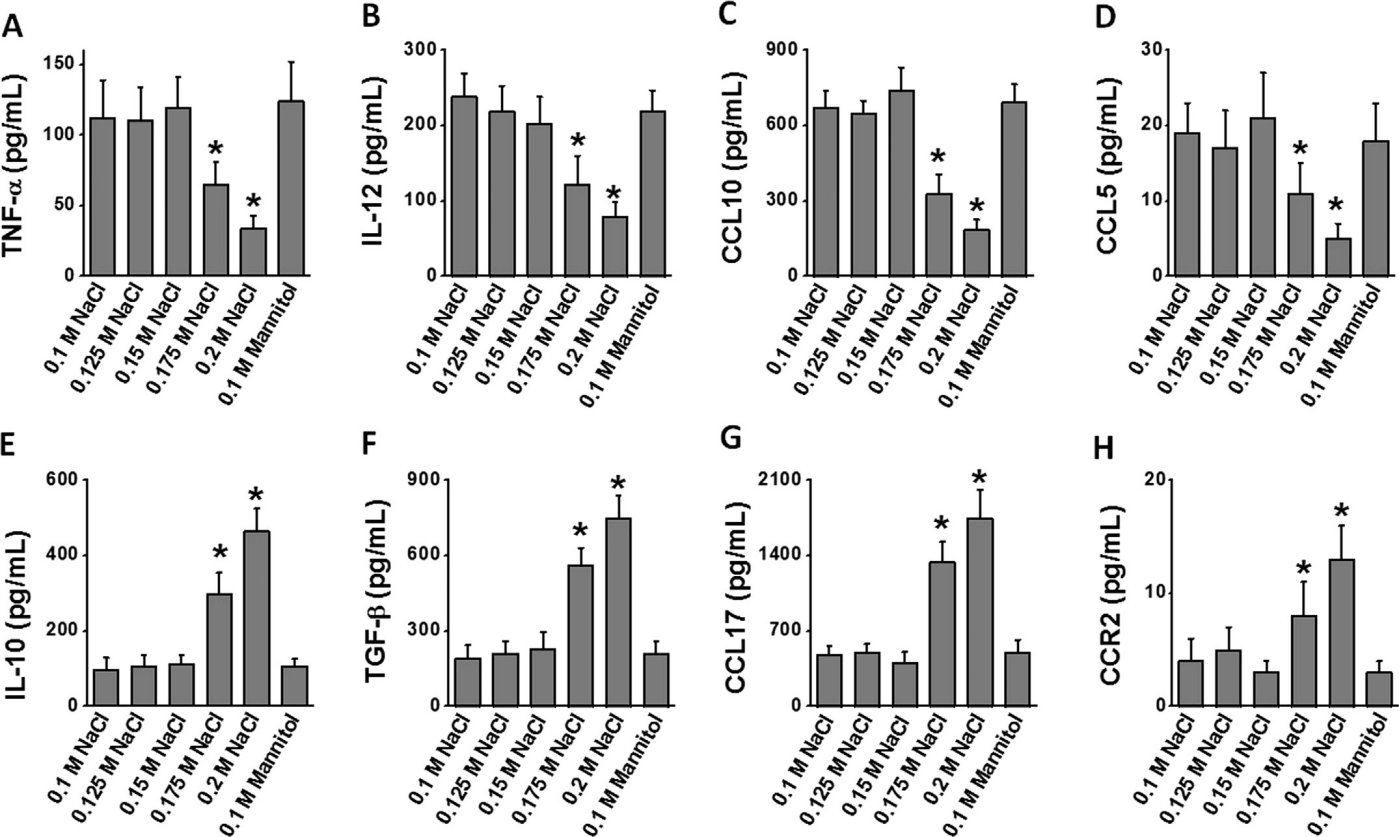
Cytokine and chemokine analysis in the supernatant or lysate collected from adherent-PBMCs following treatment with varying salt concentration (0.1–0.2 M NaCl) and equimolar mannitol (0.1 M mannitol+0.1 M NaCl). ELISA analysis of A-G) TNFα, IL12, CCL-10, CCL-5, IL-10, TGF-β, and CCL17 in the supernatant; and (F) CCR2 in the cell lysate. Data represented mean values ± SEM from five independent experiments. Student-*t*-test performed for statistical analysis (significance p<0.05).

We next studied if the cellular phenotype switch, as observed by the cell surface markers, is represented by switch in the cell phenotype specific cytokine switch. Towards this we determined the expression of various cytokines and chemokines under high salt treatment conditions. As shown in Fig. 3(A)-(D), following high salt treatment there was decrease in the MΦ1 phenotype specific pro-inflammatory cytokine and chemokine profile, as noted by decrease in TNFα (3.3 fold), IL-12 (2.3 fold), CCL-10 (2 fold) and CCL-5 ( 3.8 fold) secretion over regular (0.1 M) salt treatment. Conversely, high salt treatment has induced enhanced secretion of the MΦ2 phenotype specific anti-inflammatory cytokine, as noted by increase in IL-10 (4.8 fold), TGFβ (3.9 fold), CCL-17 (3.7 fold) and CCR-2 (4.3 fold) expression over regular (0.1 M) salt treatment. As noted earlier we have seen a trending pattern in the cytokines and chemokines from 0.15 M to 0.2 M NaCl concentrations. As expected equi-molar mannitol did not induce any change in cytokine profile, and was similar to MΦ1 phenotype. These data clearly indicate that the high salt induces a macrophage phenotype switch from pro-inflammatory MΦ1 to anti-inflammatory MΦ2 phenotype.

### 3.3 Reduced phagocytic efficiency upon high salt treatment

**Fig. 4:**
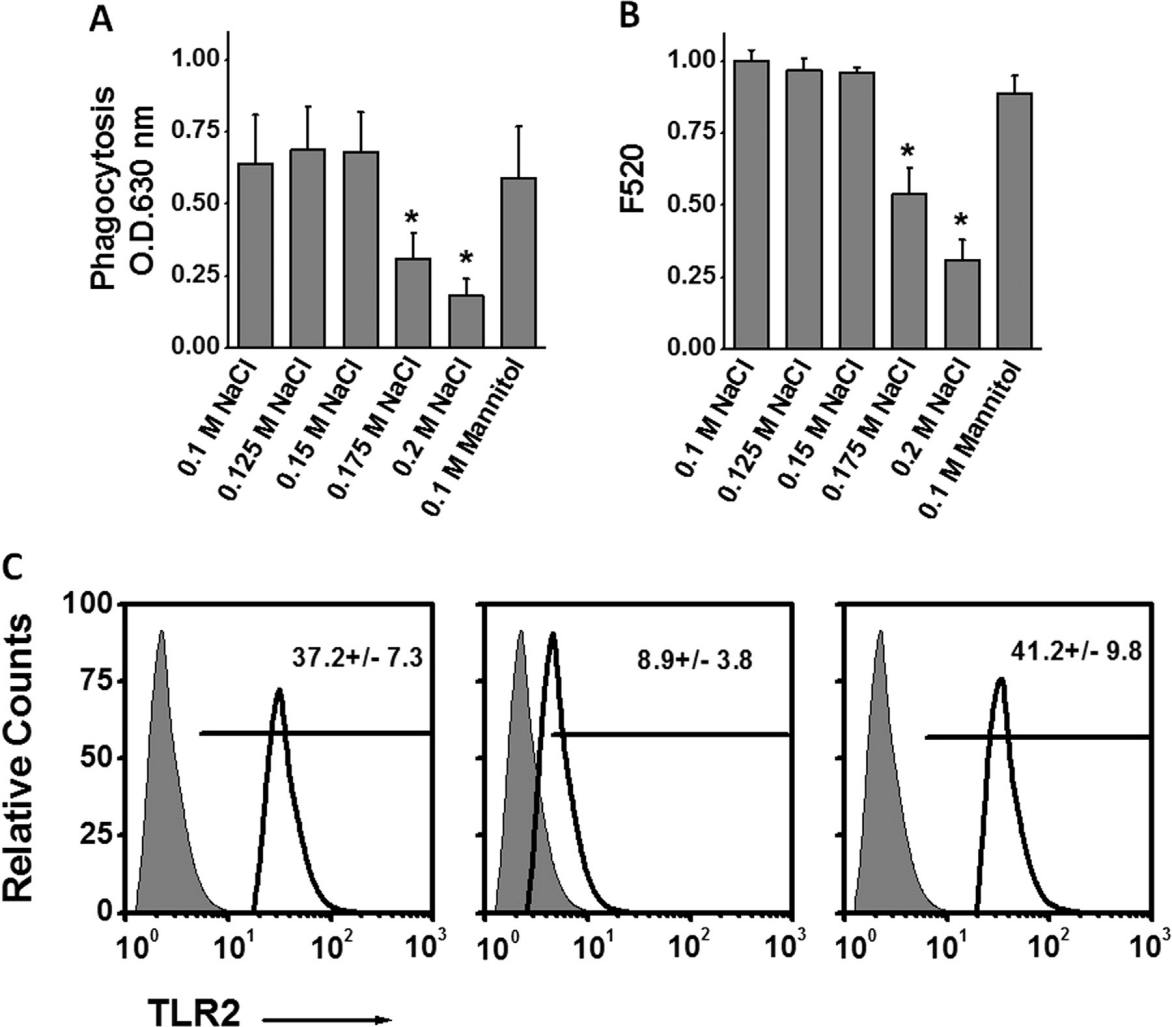
Reduced phagocytic efficiency upon high salt treatment in adherent-PBMCs following treatment with varying salt concentration (0.1–0.2 M NaCl) and equimolar mannitol (0.1 M mannitol+0.1 M NaCl). (A) EColi(-K12) bio-particle uptake fluorescence emission of FITC analyzed at 520 nm; and (B) RBC-lysis assay analyzed at 630 nm absorbance. (C) Enhanced surface expression of TLR-2 following treatment with various conditions mentioned above. Data represented mean values ± SEM from five independent experiments. Student-*t*-test performed for statistical analysis (significance p<0.05).

As our previous results demonstrated that high salt induced anti-inflammatory phenotype, and because the primary function of activated macrophages in inflammation is phagocytosis and antigen presentation, we therefore performed studies to determine the possible changes in phagocytic efficiency following high salt treatment. Towards this we have performed RBC-lysis assay (Fig. 4(A)) and EColi(-K12) bio-particle uptake (Fig. 4(B)). As determined by both of these phagocytosis assay procedures, there was a greater than 70% (p<0.05) reduction in the phagocytic efficiency of the macrophages following high salt treatment. As toll-like receptors (TLRs) are known to play a central decisive role in phagocytosis, we studied the potential changes in surface expression of TLR-2 following high salt treatment. As shown in Fig. 4(C), high salt treatment resulted in decreased TLR-2 surface expression from 37.2±7.3% to 8.9±3.8% (p<0.05). Taken together, these data clearly indicate that high salt treatment induced anti-inflammatory macrophage phenotype with reduced phagocytic and potentially antigen presentation efficiency.

### 3.4 Inhibition of iNOS activity following high salt stimulation

**Fig. 5:**
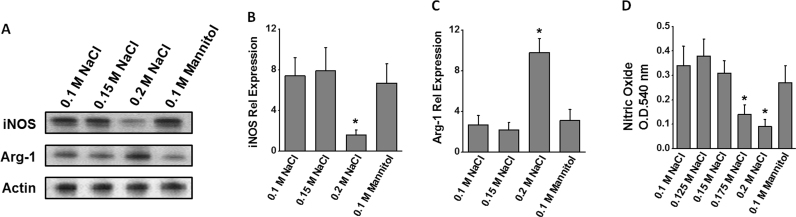
**:** Inhibition of nitric oxide pathway by high sodium chloride in adherent-PBMCs following treatment with varying salt concentration (0.1–0.2 M NaCl) and equimolar mannitol (0.1 M mannitol+0.1 M NaCl). (A) Western blot analysis of the protein expression of the enzymes iNOS and Arg-1; Quantitative RT-PCR analysis of the mRNA expression of *iNOS* (B) and *Arg-1* (C); and (D) ELISA analysis of nitric oxide in the cell lysate. Data represented mean values ± SEM from five independent experiments. Student-*t*-test performed for statistical analysis (significance p<0.05).

The inflammatory phenotype of macrophages has been attributed to its inducible nitric oxide *syn*thetase (iNOS) enzymatic activity and its ability to generate pro-inflammatory mediators, nitric oxide and reactive nitrogen species [19]. As shown in Fig. 5(A)-C, high salt treatment induced a 4.6 fold reduction in the expression of iNOS enzyme. However, there was a 3.6 fold increase in the expression of the enzyme arginase-1 (Arg-1), an anti-inflammatory enzyme a known iNOS antagonistic function. To directly evaluate the modulation of iNOS enzymatic activity, we performed nitric oxide assay. As shown in Fig. 5(D), high salt treatment resulted in reduced synthesis of nitric oxide in the macrophages, and thus strongly collaborating with the iNOS expression analysis. These data strongly support the conclusion that high salt treatment induces an anti-inflammatory macrophage phenotype.

### 3.5 Reversal of macrophage phenotype following re-treatment with regular (0.1 M NaCl) salt concentration

**Fig. 6:**
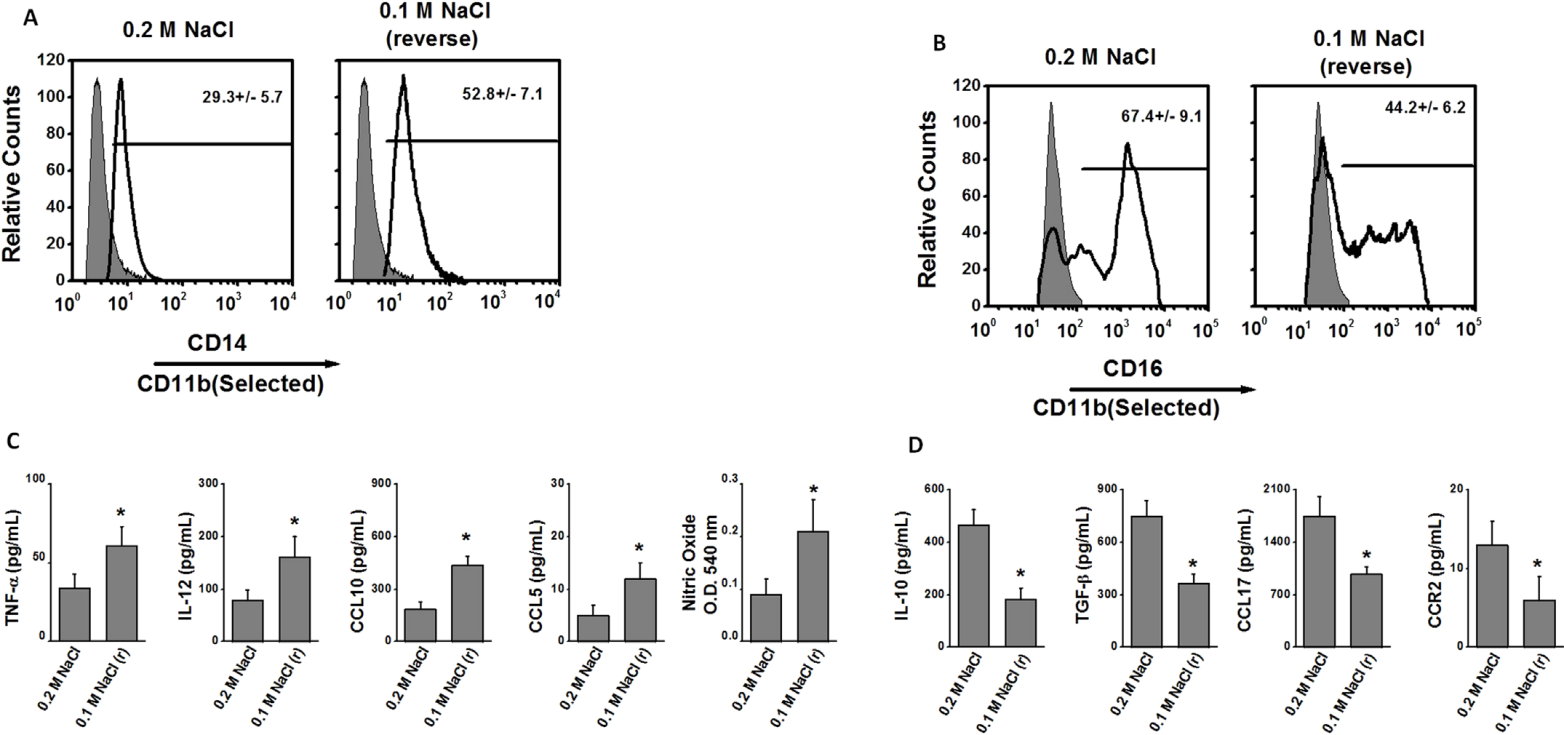
Reversal of macrophage phenotype following re-treatment with regular (0.1 M NaCl) salt concentration. The adherent macrophages previously cultured in high salt for 48 h, for another 48 h with reversing to regular media (0.1 M NaCl). The macrophage phenotype for expression of CD14 (A) and CD16 (B) in CD11b selected cells have been analyzed. (C, D) The inflammatory chemokines and cytokines (same cytokines and chemokines analyzed in Fig. 3) were analyzed for their change in expression pattern following re-treatment in regular salt media; ‘0.1 M NaCl (r)’ refers to reversal of salt concentration back to regular salt containing media in the cells pretreated with high (0.2 M NaCl) salt. Data represented mean values ± SEM from five independent experiments. Student-*t*-test performed for statistical analysis (significance p<0.05).

To study the potential effect reversal of macrophage phenotype following retreatment with regular (0.1 M NaCl) salt media, we have cultured the adherent macrophages previously cultured in high salt for 48 h, for another 48 h with reversing to regular media (0.1 M NaCl). As shown in Fig. 6, reversing the salt concentration from high (0.2 M NaCl) to regular (0.1 M NaCl) induced reveral of macrophage phenotype, as noted by, enhanced pro-inflammatory (MΦ1) phenotype from 29.3–52.8% and reduced anti-inflammatory (MΦ2) phenotype from 67.4–44.2%. Although the reversal was not completely back to the original starting point (which was MΦ1 77.4% and MΦ2 17.2%, noted in Fig. 2) there was still a significant reversal from anti-inflammatory to pro-inflammatory macrophage phenotype, following reversal of salt treatment. Similar congruent reversal to inflammatory cytokines and chemokines expression was observed (Fig. 6 C-D). These data demonstrate that the salt induction of inflammatory changes in macrophage phenotype was reversal. Further, these data suggest that in tumor micro-environment potentially salt-modified diet (such as low-salt diet) could potentially reverse the tumor associated macrophage (MΦ2-phenotype) to inflammatory MΦ1-phenotype which could be potentially helpful for tumor suppression. This, however, warrants further clinical translational studies.

## 4 Discussion

\An inflammatory component is present in the microenvironment of most tumors. Several lines of evidence have led to a generally accepted paradigm that inflammation and cancer are interlinked. Macrophages are a heterogeneous population of innate myeloid cells involved in inflammatory responses [20]. Macrophages have different functions, and different transcriptional profiles, but all are required to maintain homeostasis. Studies in mice showed that a distinct population of macrophages helps to disperse malignant cells to survive and grow in distant sites. Tumor associated macrophages (TAMs) play an important role in tumor immunity and show similar functions to MΦ2 phenotype [4]. TAMs are a polarized MΦ2 macrophage population with potent immunosuppressive functions [21]. The predominant expression of these M2 macrophages reflects the late stage of tumor progression and poor outcome. Several surface markers have been attributed to the M1/M2 phenotype and is also dependent upon the tissue microenvironment [22]. In particular, the difference in the expression of lipopolysaccharide-binding-receptor (CD14) and of Fcγ receptor III (CD16) has been used to distinguish various MO subpopulations [23]. The majority (70–90%) of peripheral blood MO consists of cells strongly CD14 positive and CD16 negative (CD14^+^CD16^-^) and are usually regarded as ‘classical activated’. The remaining MO express CD16 but have different expression levels of CD14 which are predominantly regarded as alternately activated [24]. In line with this data, our current research demonstrated that MΦ1 phenotype (CD11b^+^CD14^+^CD16^low^) accounted for 77% of the activated macrophages. However, after treatment with high salt (0.3 M NaCl) there was a 4-fold increase (Fig. 1) activated to MΦ2 phenotype (CD11b^+^CD14^low^CD16^+^). As tumors are known to have high salt micro-environment [25], our data strongly suggests that high salt induces anti-inflammatory MΦ2 phenotype which are predominantly accumulated in tumor micro-environment.

The cytokine network expressed at the tumor site plays a central role in the orientation and differentiation of recruited mononuclear phagocytes, thus contributing to direct the local immune system away from antitumor functions [26]. This idea is supported by both preclinical and clinical observations [27], [28] that clearly demonstrate an association between macrophage number/density and prognosis in a variety of murine and human malignancies. The classically activated MΦ1 phenotype, can be induced by interferon γ (IFNγ)/tumor necrosis factor α (TNFα) and exert a cytotoxic effect on cancer cells [29]. The alternatively activated TAMs, the MΦ2 phenotype, can be induced by transforming growth factor β (TGFβ) and provide a nutritional advantage for cancer cells [21]. A critical difference between MΦ1 and MΦ2 TAMs is their secretion profiles. The MΦ1 macrophages release inflammatory cytokines (e. g., IL6 and TNF) that kill cancer cells; however, MΦ2 macrophages the predominant tumor associated macrophages in late stage cancers release anti-inflammatory IL-10, and a variety of growth factors (VEGF, FGF etc), that promote growth and vascularization of the cancer mass [30]. Cancer cells often secrete MΦ2-type cytokines such as IL-10, CCL2, CXCL12, and VEGF [31]. Further, IL-10 promotes the MΦ2 alternative pathway of macrophage activation and induces TAM to express MΦ2-related functions and thus playing as a positive feed-forward cycle [32]. In this study we have shown that following treatment with high salt the newly activated adherent macrophages induce anti-inflammatory (IL-10 and TGF-β) cytokines resembling MΦ2 phenotype (Fig. 2) in the tumor associated macrophages (TAM).

Major functional properties of macrophages include phagocytosis, endocytosis, secretion, and microbial killing [33]. Because macrophages are able to perform all these activities in the steady state, MΦ1 and MΦ2 contribution to disease is, for the most part, modulation and functional tuning. While classically activated MΦ1 macrophages have been shown to have high phagocytic activity, the anti-inflammatory MΦ2 phenotype is shown to have diminished phagocytic activity. When macrophages have recruited to the tumor microenvironment, the immune phagocytic and eventual antigen presentation functions of macrophages have been suppressed and switched to MΦ2 phenotype. This phenotype switch is thought to be critical for the growth of the tumor and survival of malignant cells which subvert the adaptive immune responses resulting in tumor progression and metastasis [34]. In line with this evidence, our current study suggests that high salt potentially similar to the tumor micro-environment reduces the phagocytic efficiency (Fig. 3) of the macrophages.

The classically activated macrophages possess a markedly enhanced ability to kill and degrade intracellular microorganisms, and this is generally considered a defining functional criterion of M1- macrophage. The phagocytic function is accomplished by an increase in the production of toxic oxygen species and an induction of the inducible NO synthase (iNOS) gene to produce NO [35]. When tumor cells are already at the escape phase, they employ many pathways to maintain low levels of NO/RNS, as low amounts of NO/RNS are actually beneficial to the tumor cells. Anti-inflammatory mediators in the tumor microenvironment (e. g., TGFβ) reduce transcription of iNOS mRNA and effectively lower production of NO. Arginase-1 (Arg-1) that is highly expressed by the TAMs and MDSCs depletes l-arginine and leaves insufficient amounts of this common substrate for iNOS activity [36]. Our studies demonstrate that high salt reduces the pro-inflammatory nitric oxide release (Fig. 4) from macrophages and induces antagonistic Arg-1 expression.

Taken together, these data suggest that high salt extracellular environment induces an anti-inflammatory MΦ2 macrophage phenotype with poor phagocytic and potentially reduced antigen presentation capacity. The TAMs are known to possess anti-inflammatory, pro-angiogenic and tumor-promoting properties [37]. Reprogramming macrophages to switch their phenotype could provide stimulatory/destructive (MΦ1) or suppressive/protective (MΦ2) therapeutic strategies. In clinical trials, therapies which interfere with MΦ2 (TAM) macrophages by preventing MΦ2 macrophages from differentiating resulted in reduced tumor growth and survival [38]. Our current studies provide novel molecular basis to explain the anti-inflammatory effect of tumor infiltrating macrophages with potential beneficial effects of low-salt diet to cancer patients.

## Conflict of Interest

All authors have no conflict of interest.
